# Influence of a Crosslinker Containing an Azo Group on the Actuation Properties of a Photoactuating LCE System

**DOI:** 10.3390/polym8120435

**Published:** 2016-12-14

**Authors:** Lukas B. Braun, Torsten G. Linder, Tristan Hessberger, Rudolf Zentel

**Affiliations:** Institute of Organic Chemistry, Johannes Gutenberg-Universität Mainz, D-55099 Mainz, Germany; l.braun@uni-mainz.de (L.B.B.); tolinder@students.uni-mainz.de (T.G.L.); hessberger@uni-mainz.de (T.H.)

**Keywords:** liquid crystalline elastomers, photoactuation, polymer film, light-responsive, azo, crosslinker, actuator, vis photo polymerization, liquid crystalline network

## Abstract

Photoactuating liquid crystalline elastomers (LCE) are promising candidates for an application as artificial muscles in microdevices. In this work, we demonstrate that by optimizing (1) the illumination conditions and (2) the mixture of azo monomer and azo crosslinker, thick films of an all-azo LCE can be prepared, which show a strong length change without bending during photoactuation. This becomes possible by working with white light (about 440 nm), whose absorption is low, leading to a large penetration depth. By adding an azo crosslinker to a previously prepared system, several improvements of the actuation properties—like a stronger photoactuation at lower operational temperatures—could be achieved. In addition, films of different crosslinker concentrations and thicknesses were produced by photopolymerization at varying temperatures within a magnetic field, and their thermo- and photoresponsive behavior was investigated. An extraordinarily strong maximal thermal actuation of 46% and—by exposure to white light at 70 °C—a photoresponsive change in length of up to 40% in just about 13 s could be obtained. Even densely crosslinked samples were still able to photoactuate remarkably. Isothermal back-deformation could either be achieved by irradiation with red light (7 min) or by keeping the film in the dark (13 min).

## 1. Introduction

Liquid crystalline elastomers (LCE) are well known as soft actuators and are promising candidates for an application as artificial muscles in microrobotics [1,2,3,4,5,6,7,8,9]. First predicted by de Gennes in 1975 [10], these materials combine the entropy elasticity of elastomers with the anisotropic properties of the liquid crystalline phase. The liquid crystalline state is a mesophase between the highly ordered crystalline and the disordered isotropic state, in which the so-called mesogens are all aligned along a common direction, named the director. In LCEs, these mesogens are attached to a slightly crosslinked polymer network either via an alkyl spacer (side-chain LCEs) or they are incorporated into the polymer chain (main-chain LCEs), with the latter usually showing the stronger actuation [11,12]. Below the clearing temperature, the polymer chains experience the anisotropic environment of the liquid crystalline phase and adopt an anisotropic chain conformation [13,14], which may be stretched (as in the example presented below). Thereby, the magnitude of chain anisotropy parallels the magnitude of the nematic order parameter [14]. If the sample is heated above the phase transition temperature, the nematic order is lost and the polymer backbone can relax into the more favored random-coil conformation. This leads to a macroscopic deformation of the whole sample. So, LCEs change their dimensions both within the liquid crystalline phase, as the order parameter varies with temperature, and especially at the phase transition temperature. However, this can only be observed macroscopically if the sample is present as a monodomain, which can be achieved by applying external forces during the crosslinking step. For polymer films, a uniform orientation of the single domains can, for example, be obtained when the prepolymerized sample is stretched, with the aid of a photoalignment layer or by application of an electric or magnetic field [15,16,17,18]. LCE particles and fibers with a good orientation of the mesogens can also be fabricated in a microfluidic device [19,20,21,22].

**Figure 1:**
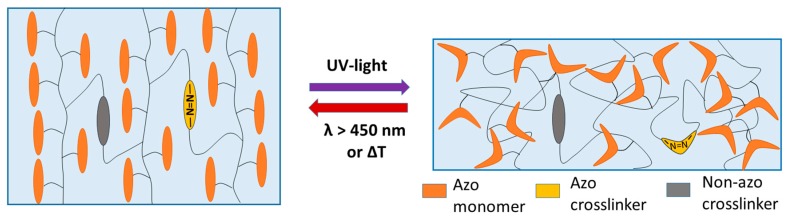
Schematic drawing of an azo-liquid crystalline elastomer’s (LCE’s) photoactuation with an azo and a non-azo crosslinker. Compared to the latter, the azo crosslinker can contribute to the chemical modification of the LC material.

LCEs that can be triggered by stimuli other than external temperature variation have been developed as well. Here, other methods to change the order parameter of the liquid crystalline phase are used. This includes swelling in a good solvent [23], moisture [24], and indirect heating by magnetic [25,26] or electric fields [27]. Due to the good control over its intensity and the ability to accurately trigger a sample without physically touching it, light as stimulus is especially attractive [28,29,30,31,32,33,34,35]. Examples of UV-, vis-, and near infrared (NIR)-photoactuation are known from literature [36,37,38,39,40,41]. In most cases, the photoactuation is a result of a chemical modification of the LC material by photoisomerization. LCEs containing an azo-benzene group in the mesogenic unit are attractive for this purpose. Irradiation of this moiety with UV light drives *trans*- to *cis*-isomerization and thus causes an isothermal shift of the phase transition temperature due to the formation of non-mesogenic compounds (Figure 1) [14]. As a result, photoactuation occurs when azo-LCEs are exposed to UV light close to the phase transition temperature. This process can be reversed either by keeping the sample in the dark (thermal back-isomerization) or by irradiation with light of higher wavelength (λ > 450 nm, photochemical back-isomerization) [42,43,44,45]. Also, the physics of the deformation induced by light are described in the literature, but are out of the scope of this article [14,46,47,48].

Recently, we prepared an azo-LCE system, which showed a fast and strong photoactuation (length change) of up to 34%, when illuminated with a white light-emitting diode (LED) lamp at 440 nm [49]. This is remarkable as, firstly, most azo-containing LC networks are densely crosslinked, which limits their length change. Secondly, and more importantly, usually bending movements are reported. These bending movements are—besides some carefully designed experiments to promote bending by the director pattern—mostly the result of a strong variation of the light intensity inside the LC network, due to the high optical density of the azo system in the UV region. Thus, concepts to increase the optical penetration depths in weakly crosslinked systems are required in order to obtain “thick” LCEs with large optically induced length changes without bending. In addition to these considerations, it is desirable to induce photo-addressable crosslinking points (Figure 1) because they allow a variation of the crosslinker density without strongly changing the optical properties of the LCEs. In addition, former studies claim that, by using azo-benzenes as the crosslinker, a higher mechanical response of LCE films can be achieved than by just having the azo moiety incorporated in the mesogenic side groups [50,51]. A scheme of such LCE systems is drawn in Figure 1.

## 2. Materials and Methods

Butyryl choline butyltriphenylborate (Borate V) was purchased from Spectra group limited, Inc. (Millbury, OH, USA). All other chemicals and solvents were purchased from Sigma-Aldrich (Sigma-Aldrich Chemie GmbH, Taufkirchen, Germany) or Arcos (Geel, Belgium). The liquid crystalline azo monomer (4-butoxy-2′-(4-methacryloyloxybutoxy)-4′-(4-butoxybenzoyloxy)azobenzene) [52], the non-azo crosslinker (1,4-*bis*(4-(6′-acryloxy-hexyloxy)benzoyloxy)-2-toluene) [53], the azo crosslinker (4,4′-*bis*[9-(acryloyloxy)nonanyloxy]azobenzol) [54] and the dye for the initiator (1,3,3,1′,3′,3′-hexamethyl-11-chloro-10,12-propylene-tricarbocyanine iodide) [55] were synthesized according to the literature. A red light HighPower LED (623 nm) was purchased from Conrad (Hirschau, Germany), and the cold light source KL 1600 from Schott (Mainz, Germany).

### 2.1. Preparation of Polymer Films

The monomer mixture was prepared by dissolving the monomer, the azo crosslinker (varying concentration), 1.4 wt % 1,3,3,1′,3′,3′-hexamethyl-11-chloro-10,12-propylene-tricarbocyanine iodide and 3 wt % Borate V in dichloromethane, followed by distillation of the solvent. A few crystals of the resulting solid were melted at 90 °C on a glass slide, which was lying on a precision hot plate (Harry Gestigkeit GmbH, Düsseldorf, Germany) within the magnetic field of a horseshoe magnet (*B* > 100 mT). A second glass slide was put on top of the lower one, separated by spacer foil of varying sizes. After cooling the sample to the desired polymerization temperature, it was irradiated with red light of the HighPower LED for about 1 min. Afterwards, the two glass slides were separated with a scalpel, with the film just sticking to one of them. In order to investigate the actuation properties of the film, little pieces were cut out of the LCE film.

### 2.2. Characterization

UV–vis spectra were recorded using the spectrophotometer V-630 (Jasco Labor-u. Datentechnik GmbH, Groß-Umstadt, Germany) with dichloromethane being the solvent (*c* = 50 µmol/L). Here, for the irradiation experiments, the sample inside the cuvette was irradiated with an Oriel LSH302 (500 W) lamp (Newport Spectra-Physics GmbH, Darmstadt, Germany) equipped with a band filter (323–385 nm), and with a cold light source KL 1600, for 7 min, respectively. The transmittance spectrum was recorded with the same spectrometer. In order to simulate the transmittance of an LCE film with a thickness of 50 µm, the concentration of the solution was chosen to be 80 mmol/L so that the light beam passed through as many azo groups as it would in the film. The actuating properties of the LCE films were investigated under the light microscope Olympus BX51 (Olympus Deutschland GmbH, Hamburg, Germany) equipped with a hot-stage (Linkam TMS 94, Linkam Scientific Instruments, Waterfield, Tadworth, UK) and the camera Olympus ColorView II. In order to measure the length of the films, the imaging software cell^D (Olympus Deutschland GmbH, Hamburg, Germany) was used. For the irradiation experiments, the sample was heated to the desired temperature and irradiated with the cold light source until no further deformation could be observed. Two light guides were used to focus the light upon the sample, giving a light spot of about 1 cm in diameter. A red-light filter (OG 590 Schott, for spectrum see Supplementary Materials, Figure S1) was held between the light guides and the sample in order to investigate the photo-induced back-deformation of the film.

## 3. Results and Discussion

### 3.1. Selection of the LCE System

**Figure 2:**
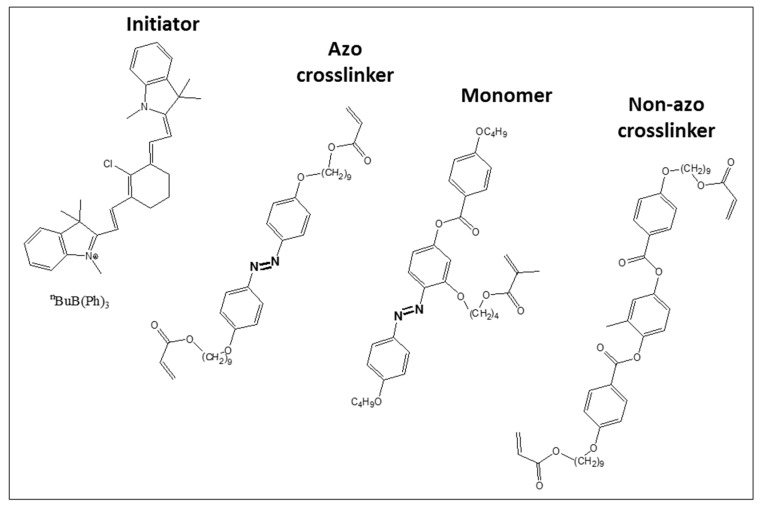
Chemical structures of the initiator (λ_abs_ = 780 nm), azo crosslinker (c 74 °C n 91 °C i), monomer (c 53 °C n 84 °C i), and the non-azo crosslinker (c 66 °C n 92 °C i). Note: c = crystalline, n = nematic, i = isotropic.

As mentioned in the introduction, we recently prepared a novel azo-LCE system, which showed an extraordinary strong photoactuation of up to 34% at 70 °C. However, since the crosslinker (non-azo crosslinker, Figure 2) did not bear an azo group, highly crosslinked samples showed a weak change in length compared to the thermal actuation. For an application in mechanical devices, high crosslinker concentrations are desirable because they provide the necessary stability. Hence, we chose to use an azo crosslinker for this work, which also is shown in Figure 2, along with the monomer and initiator. Compared to the three benzene rings there, the new crosslinker just contains two benzene rings in the mesogenic unit. So, the best irradiation temperature should be lower, caused by a reduction of the clearing temperature. A lower actuation temperature would make the LCE better applicable in mechanical devices. Furthermore, we expect an even higher photoactuation of this system, since the crosslinker can contribute to the reduction of the mesogens’ orientation.

The photoresponsive crosslinker (crystalline 74 °C nematic 91 °C isotropic) is known from literature already. There it was used, however, in a rather densely crosslinked system, where dimensional variations are limited and the actuator works mostly on bending [30]. Compared to the monomer, this molecule has a larger separation between the *trans*- and *cis*-band, and thus can be switched a bit more efficiently between both conformers compared to the monomer (see Supplementary Materials, Figures S2 and S3). By exposure to UV light, the π→π*-band of the *trans*-isomer (λ_max_ = 365 nm) disappears and the *cis*-isomer’s n→π*-band (λ = 450 nm) shows up. This process is reversible by irradiation with red light (λ > 500 nm). However, an excitement at the maximum of the *cis*-band itself does not lead to a reversion, as the *trans*-isomer still absorbs light strongly at this wavelength. 

**Figure 3:**
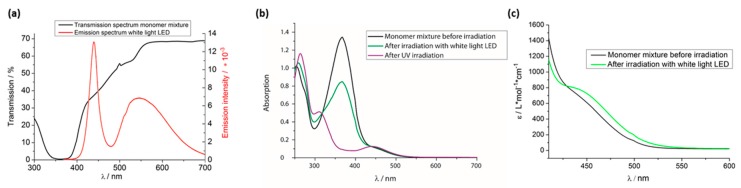
(**a**) The crosslinker’s transmission spectrum compared to the emission spectrum of the white light light-emitting diode (LED) which was used for the irradiation; (**b**) UV–vis spectrum of the monomer mixture before and after irradiation with UV light as well as after irradiation with the white light LED; (**c**) molar extinction coefficient spectrum of the monomer mixture before and after irradiation with the white light LED. All absorption/transmission spectra were measured at room temperature (RT) in dichloromethane (DCM).

To improve this system from the photochemical side, we started our considerations with the fact that the actuation reported is nearly always a bending, which is a result of the high optical density of the “concentrated” azo system between 300 and 400 nm. This leads to the fact that the photon flux gets reduced over the thickness of the film and the side exposed to the light source is exposed to a much higher light intensity compared to the back side. In our case, a film of the thickness we are aiming at would have a transmission of only about 0% (see Figure 3a).

Now, we wanted to prepare a photoactuating elastomer, which offers (1) large length changes for (2) thick samples (necessary to create enough mechanical force), and (3) without bending.

To obtain a large deformation, the amount of crosslinker has to be reduced. That is relatively easy (it was also done in the recent paper [42]), because most other systems work with close to 100% of crosslinker. The main problem concerning this task is how to reduce the optical density of the azo system (see Figure 3a) so that even thick samples experience an “about uniform” exposure to light, because this is necessary to prevent bending. In this context, we recognized that nearly all the work on azo systems focuses on the maximal absorption of the *trans*-azo chromophore at 360 nm, which is the reason for the high optical extinction. On the other side, the wavelength region between 400 and 450 nm (Figure 3a) offers much less extinction (lower optical density), while the absorption of the *trans* form is below 440 nm, but still higher than that of the *cis* form (Figure 3c). This wavelength region is now easily accessible with blue LEDs (Figure 3a). So, it seems possible to induce a *trans*–*cis* photoisomerization by exposing the azo-containing LCE to light of 400–450 nm. This isomerization is less effective than illumination at 360 nm, so that we get less *cis*-isomer on illuminating the LC mixture (see Figure 3b and Supplementary Materials, Figures S2 and S3 for the pure compounds). However, our system is also composed of 100% azo mesogens.

As the initiator for the photopolymerization, we chose 1,3,3,1′,3′,3′-hexamethyl-11-chloro-10,12-propylene-tricarboxcyanine triphenylbutyl borate (CBC) which was already used by Keller et al. [56]. With an absorption maximum of 780 nm, the polymerization can be initiated by red- or NIR-light, thus the wavelength is high enough not to cause any *trans*–*cis* isomerization and not to be absorbed by the monomer before it can reach the initiator molecules. Since the decay product of CBC still absorbs light at the same wavelength, we synthesized it in situ by adding the initiator’s dye (with iodide as counterion) and an excess of the borate (as ammonium salt) to the monomer mixture. The latter also acts as a bleaching agent for the dye so that even thick samples can be cured. The mechanism of the initiation step is already discussed in our previous work [49].

### 3.2. Investigation of the Best Preparation Parameters

To optimize the film production parameters of our system, we synthesized LCE films at different polymerization temperatures as well as with varying crosslinker ratios and sample thicknesses. Therefore, inside the magnetic field of a horseshoe magnet (*B* > 100 mT), a few crystals of the monomer mixture were melted on a glass slide and heated to the isotropic phase at 90 °C. In former studies, it could be shown that a magnetic field of this strength is already sufficient to provide a good orientation of the mesogens inside a liquid crystalline phase [57]. A second glass slide was put on top of the first one, separated by spacer foil of varying sizes, before the sample was cooled to the liquid crystalline phase (*T*_NI, cooling_ = 67 °C) and polymerized with red light (623 nm, 1 min). In order to determinate the actuation properties, we cut little pieces of the LCE film and investigated their change in width as a response to the stimulus under the polarized light microscope (POM). To measure the thermoresponsive actuation, the film was heated above the clearing temperature on a precision hot plate, and the photoresponsive actuation was measured by irradiating it with a cold light source (continuous wavelength, for spectrum see Figure S1) at 70 °C. The actuation was then calculated by dividing the width of the film before the deformation (*w*_0_) by the width afterwards (*w*_1_). (Actuation = 100% × *w*_0_/*w*_1_).

**Figure 4:**
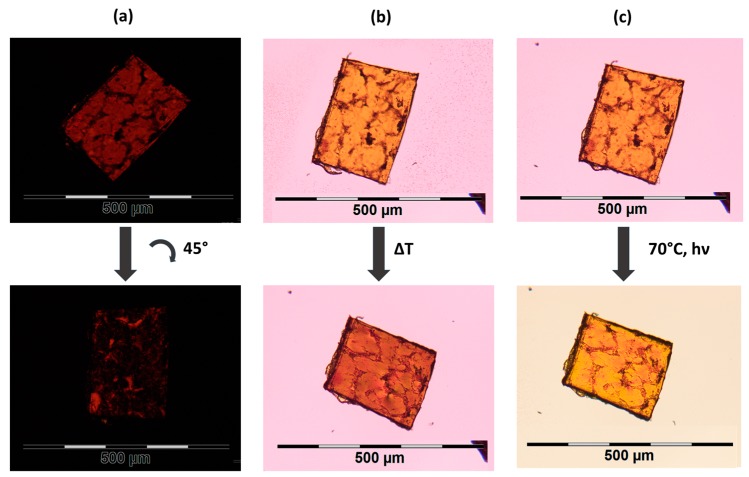
Polarized light microscope (POM) images of an LCE film with 10 mol % crosslinker, a thickness of 50 µm, and polymerized at 60 °C. (**a**) Cross-polarized light image at 70 °C with the film being rotated by 45° in the second image; (**b**) film in its glassy phase at RT and after heating it above the clearing temperature at 110 °C; (**c**) film at 70 °C before and after illumination with a white cold light source.

Figure 4 shows one of the produced films (10 mol % crosslinker, thickness: 50 µm, polymerized at 60 °C) under the polarized light microscope. According to Figure 4a, the mesogens are well oriented since the birefringence disappears by rotating the film by 45°. In Figure 4b, the thermal actuation can be seen. By heating the film from room temperature to above the nematic-isotropic phase transition temperature (*T*_NI_), it becomes much shorter, simultaneously widening and thickening. *T*_NI_ was found to be about 92 °C according to the POM measurements. A slightly weaker actuation (photoactuation) is observable when the sample is irradiated with white light of a cold light source at temperatures close to *T*_NI_ (Figure 4c and Video S1 in Supplementary Materials).

#### 3.2.1. Influence of the Polymerization Temperature

**Figure 5:**
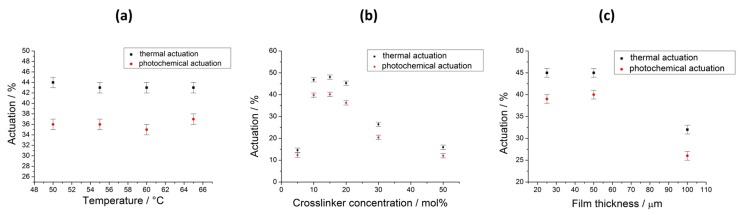
Photo- and thermal actuation of LCE films with variation of the synthesis parameters. (**a**) Influence of the polymerization temperature on the actuation for a film with 20 mol % crosslinker and a thickness of 50 µm; (**b**) influence of the crosslinker concentration (polymerization temperature: 60 °C, sample thickness: 50 µm), and (**c**) influence of the sample thickness (polymerization temperature: 60 °C, 10 mol % crosslinker concentration).

First, the influence of the polymerization temperature on the actuation properties was investigated based on a film with 20 mol % crosslinker concentration and a thickness of 50 µm. A deformation of the film was just observable for temperatures between 50 and 65 °C—the temperature range of the liquid crystalline phase—because below this temperature the mixture started to crystallize and above *T*_NI_ the orientation of the sample was lost. However, the polymerization temperature did not have a big influence on the actuation properties in this range, as can be seen in Figure 5a. For all temperatures, the thermal actuation is about 44% and the photoactuation amounts to about 36%. This can be explained by the fact that the magnetic field orients the director field (makes it more or less uniform), but it does not modify the order parameter in the LC domains. On the other side, the director field is still free to change with temperature in the LCEs, which is the basis for their deformation. In this respect, samples prepared at different crosslinking temperatures, but with about an equally oriented director field, can be assumed to behave similarly.

#### 3.2.2. Influence of the Crosslinker Concentration

The crosslinker concentration has, however, a big influence on the actuation properties of the LCE film (Figure 5b). Here, films with a thickness of 50 µm were synthesized by photocrosslinking at 60 °C. A small concentration of just 5 mol % crosslinker is obviously not enough to prepare a well-crosslinked system and leads only to a weak thermoresponsive actuation of just 15%. On the contrary, LCE films with a concentration of 10–20 mol % of crosslinker show a much stronger thermal actuation, with a change in length of about 46%. Higher concentrations of crosslinker lead then again to a decreasing actuation caused by a higher stiffness of the densely crosslinked material. The photoactuation shows a similar curve, with a maximum of 40% for crosslinker concentrations of 10 and 15 mol %. Here, for this LCE system, the ratio between thermo- and photoresponsive actuation is about the same for all crosslinker ratios.

#### 3.2.3. Influence of the Film Thickness

The influence of the film thickness was investigated as a third parameter of the LCE fabrication (Figure 5c). For this purpose, a film with 10 mol % crosslinker was polymerized at 60 °C. A strong thermal actuation of 46% was observed for a thickness of 25 µm, however, the photoresponsive actuation was—with a value of 32%—much lower. This was probably caused by bending of the film during irradiation, which made the measurement of the film’s real change in length impossible. Both, a high photo- (40%) and thermoresponsive (45%) actuation was obtained for a film thickness of 50 µm, but the actuation for both stimuli decreased again with an increasing film thickness.

#### 3.2.4. Optimum Preparation Parameters and Advantage of This System

By investigating the optimum preparation parameters, the best film was found to be produced at 60 °C, with a crosslinker concentration of 10 mol % and with a thickness of 50 µm. As mentioned in the introduction, usually a compromise has to be found for the azo concentration in photoresponsive LCEs. The results in Figure 5 prove that this is not valid for our LCE system. Here, the actinic light can penetrate through the whole sample, although every mesogen and every crosslinker unit bear an azo group. Even films with up to 100 µm thickness can be actuated uniaxially without just showing bending. This makes a very strong photoresponse of 40% possible, which is stronger than what we could find in regular journals in the literature.

### 3.3. Thermoresponsive Behavior

**Figure 6:**
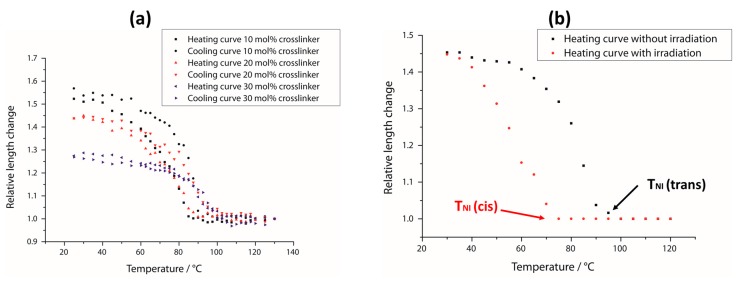
Thermoresponsive behavior of this LCE system. (**a**) Heating and cooling curves for three films with different crosslinker concentrations, respectively; (**b**) heating curve for an LCE film with and without irradiation. Here, *T*_NI_ (*trans*) is the phase transition temperature of the film’s all-*trans* state and *T*_NI_ (*cis*) the one for its photostationary state under irradiation.

To investigate the thermoresponsive behavior of the system, heating and cooling curves of an LCE film of 50 µm thickness were measured for three different crosslinker concentrations. In Figure 6a, the actuation is plotted against the temperature, resulting in s-shaped curves. As known from literature, the cooling curve is slightly shifted compared to the heating curve so that a hysteresis is obtained. Starting from low temperatures, the order parameter of the liquid crystalline phase decreases the warmer the sample becomes, and a small deformation can be observed. Close to *T*_NI_, a drastic loss of the orientation occurs so that the width of the film changes strongly by just heating it a few degrees. Above the clearing temperature, the film is in its isotropic state and thus no further actuation can be achieved. It can be seen that for higher crosslinker ratios, not only does the actuation decrease, but also the broadness of the hysteresis becomes smaller. 

By exposure to white light, the *trans*–*cis* isomerization of the azo-benzene groups is initiated in agreement with Reference [14] and a non-mesogenic compound is created, which disturbs the liquid crystalline ordering. As a result, the clearing temperature of the liquid crystalline phase is shifted to lower temperatures. Figure 6b indicates a remarkable reduction of *T*_NI_ by about 25 °C for this system. This is remarkable, as the optical measurements in Figure 3 demonstrate that under white-light illumination, only small amounts of *cis*-isomers are formed. To make sure no light-induced heating disturbed the measurement, we stopped to irradiate the sample a few seconds before, respectively. As the temperature range (in which thermal actuation happens) is larger than 25 °C, the photoresponsive actuation must be smaller than the thermal one for all temperatures, which explains the results in Section 3.2.

### 3.4. Photoresponsive Behavior

**Figure 7:**
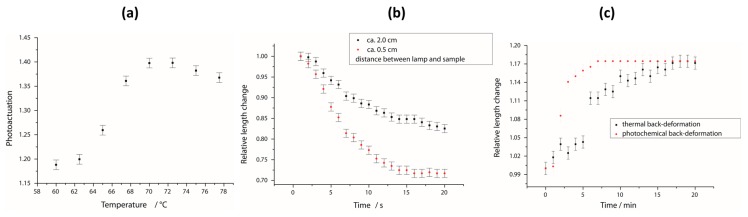
Photoresponsive behavior of this LCE system. (**a**) The photoactuation of an LCE film for different temperatures during the irradiation; (**b**) time dependence of the photoactuation for two different light intensities (distances between lamp and sample); (**c**) time dependence of the back-deformation of the sample, induced either by red light or by keeping the film in the dark (isothermal back-deformation).

In order to investigate the films’ photoresponsive behavior, the sample (thickness: 50 µm, 10 mol % crosslinker) was irradiated with a cold light source—to prevent additional heating by the lamp—and two light guides were used to focus the irradiation upon the sample, giving a round spot. First, the photoactuation was examined for different irradiation temperatures, with the results shown in Figure 7a. Photoactuation is the result of a *T*_NI_-shift by 25 °C to a lower temperature, caused by the photochemical creation of *cis*-isomers (see Figure 6b). So, for temperatures far below the phase transition temperature, just a small reduction of the LC-order parameter can be achieved, resulting in a weak actuation. By irradiating the film at temperatures close to *T*_NI_, the sample transfers into the isotropic state and the orientation is lost completely. Thus, a strong actuation can be obtained, which reaches its maximum at 70 °C. Irradiation of the sample above *T*_NI_ leads to no response since the orientation is lost already. These results are in good accordance with Figure 6b, in which the biggest gap between both heating curves is around 70 °C.

In Figure 7b, the photoactuation is plotted against the irradiation time for two different light intensities. In either case, an almost linear drop of the curve is obtained that flattens after a few seconds, and no further deformation can be observed after about 14 s. Here, it can be seen that the photoactuation is strongly dependent on the light intensity. While a photoactuation of about 30% was achieved for a distance of 0.5 cm between the light guide and sample, it was almost halved for a distance of 2.0 cm. Since the light source has a continuous spectrum and not all wavelengths contribute to the *trans*–*cis* isomerization of the azo compounds, no absolute values can be given for the light intensity. However, with respect to the inverse-square law, the intensity for a distance of 2.0 cm can be calculated as 6.25% of those with 0.5 cm. This drastic loss of photoactuation for lower irradiation intensities is in good accordance with the literature [56].

Furthermore, the back-deformation of the LCE with respect to two stimuli was investigated. As described in the introduction, the back-isomerization of azo-benzenes can be initiated either by irradiation with light of higher wavelengths (>500 nm) or by waiting. When the film is kept in the dark at the irradiation temperature, it slowly deforms back until it reaches the original shape after about 13 min (Figure 7c). By irradiation with red light, this process can be accelerated to 7 min. Therefore, a red-light filter (>590 nm, for spectrum see Figure S1, Supplementary Materials) was held between the light source and the sample while it was kept at the same temperature.

Summarized, we produced LCE films which show a strong thermoresponsive actuation of up to 46% by heating them above the clearing temperature and which also show a very strong photoresponsive actuation of up to 40% by irradiating them with light of a white cold light source. This process can also be reversed either by keeping the films in the dark for a few minutes or by irradiating them with red light.

### 3.5. Comparison with a Non-Azo Liquid Crystalline Crosslinker

**Figure 8:**
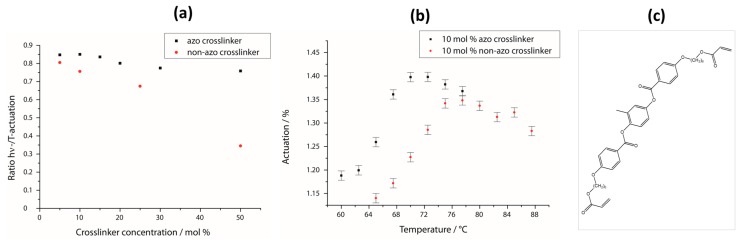
Comparison of the azo crosslinker with a similar non-azo crosslinker. (**a**) Ratio between photo- and thermal-actuation as a function of the crosslinker concentration, respectively; (**b**) comparison of the temperature dependence of both systems’ photoactuation; (**c**) chemical structure of the non-azo crosslinker.

As mentioned before, we recently published a similar system, but with a non-azo crosslinker (Figure 8c). There, the photoactuation became very small for highly crosslinked films since the crosslinker could not take an active part in the reduction of the phase transition temperature. However, for an application in devices, a high crosslinker concentration is necessary to provide good stability of the actuator. Here, by using an azo crosslinker, this issue could be overcome (Figure 8a). Weakly crosslinked films with a crosslinker concentration of just 5 mol % show a photoactuation almost as strong as the thermal actuation for both systems. By increasing the sample’s crosslinking density of the non-azo crosslinker system, however, the photoactuation becomes much weaker compared to the thermal actuation, whereas it hardly decreases for the system with azo crosslinker. So, a film with 50 mol % azo crosslinker still has a ratio of 75%, while it is strongly reduced to 30% for the one without a light-responsive group. Thus, we succeeded in producing a highly crosslinked LCE-film which still showed strong photoresponsive behavior. 

Two further advantages of the azo crosslinker system are depicted in Figure 8b. Compared to the maximal achievable photoactuation of the non-azo crosslinker system of 35%, a stronger light-induced length change of up to 40% is obtained in this case. Moreover, the curve of the photoactuation’s temperature dependence is shifted to lower temperatures, which makes this system more practical, since the device does not have to be heated up as much.

## 4. Conclusions

In this work, we demonstrate that by optimizing (1) the illumination conditions and (2) the mixture of azo monomer and azo crosslinker, thick films of an all-azo LCE can be prepared, which show a strong length change without bending during photoactuation. This becomes possible by working with white light (about 440 nm), whose absorption is low, leading to a large penetration depth. By adding an azo crosslinker to a previously prepared system, several improvements of the actuation properties—like a stronger photoactuation at lower operational temperatures—could additionally be achieved.

In detail, the best preparation parameters for this system were investigated and the maximal thermal actuation was found to be 46%. The maximal photoactuation at 70 °C irradiation temperature was—with a value of 40%—just slightly smaller. A temperature shift of about 25 °C for the nematic-to-isotropic phase transition temperature was determined by comparing the heating curves of an irradiated and nonirradiated film. Being completed after about 13 s, the photoactuation was very fast for this system, but also strongly dependent on the light intensity. Back-deformation of the sample could either be induced by keeping the film in the dark for about 13 min or it could be accelerated to just 7 min by exposure to red light (>590 nm). A comparison to the previously prepared system revealed a much better photoactuation for highly crosslinked samples, a better photo- and thermal-actuation in general, and a decrease of the best actuation temperature.

Concerning photoactuation of thick LCE samples with negligible bending, the main result of this study is the recognition of a (frequency-dependent) cutoff for photoactuation. If the most efficient absorption wavelength of *trans*-azo is used, it is possible to get the highest amount of *cis*-isomer during steady-state illumination. This will lead to the strongest reduction of the clearing temperature and it might completely destroy the LC-phase (e.g., as in our case of a system with 100% of azo-mesogens). Under these conditions the largest actuation is possible, but this system is limited to very thin films to keep the light intensity constant over the thickness of the sample and to prevent bending.

If we use less efficient wavelengths (as here at 440 nm) the *trans*–*cis* isomerization is smaller (see Figure 3) and we are limited in the reduction of the clearing temperature. However, if the overall extinction is much smaller, we can use thick samples, which will deform with negligible bending. This allows us to create more mechanical strain. Thus, we succeeded in improving the applicability of photoresponsive LCE actuators, and took the next step to application in microdevices.

## Supplementary Materials

The following are available online at www.mdpi.com/2073-4360/8/12/435/s1, Figure S1: Transmission spectrum of the red light filter OG 590 (Schott), Figure S2: UV–vis spectra of the monomer before irradiation and after irradiation with the white light LED as well as with UV light, Figure 3: UV–vis spectra of the crosslinker before irradiation and after irradiation with the white light LED as well as with UV light; Video S1: Photoactuation of an LCE film at 70 °C during irradiation with a white cold light lamp (KL1600).
