# Supplementary material for: Influence of a Crosslinker Containing an Azo Group on the Actuation Properties of a Photoactuating LCE System
Source: Polymers (Basel). 2016 Dec 14;8(12):435. doi: 10.3390/polym8120435 (PMC6432154; doi:10.3390/polym8120435)
Supplement: Supplementary file 1 [file polymers-08-00435-s001.zip › polymers-158688-suppl.pdf]

Supplementary Materials: Influence of a Crosslinker Containing an Azo Group on the Actuation Properties of a Photoactuating LCE System

Lukas B. Braun, Torsten G. Linder, Tristan Hessberger and Rudolf Zentel

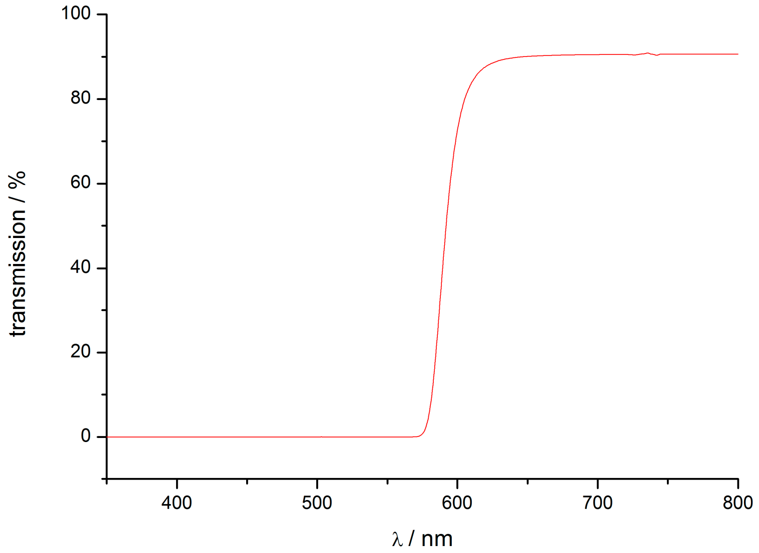

Figure S1. Transmission spectrum of the red-light filter OG 590 (Schott).

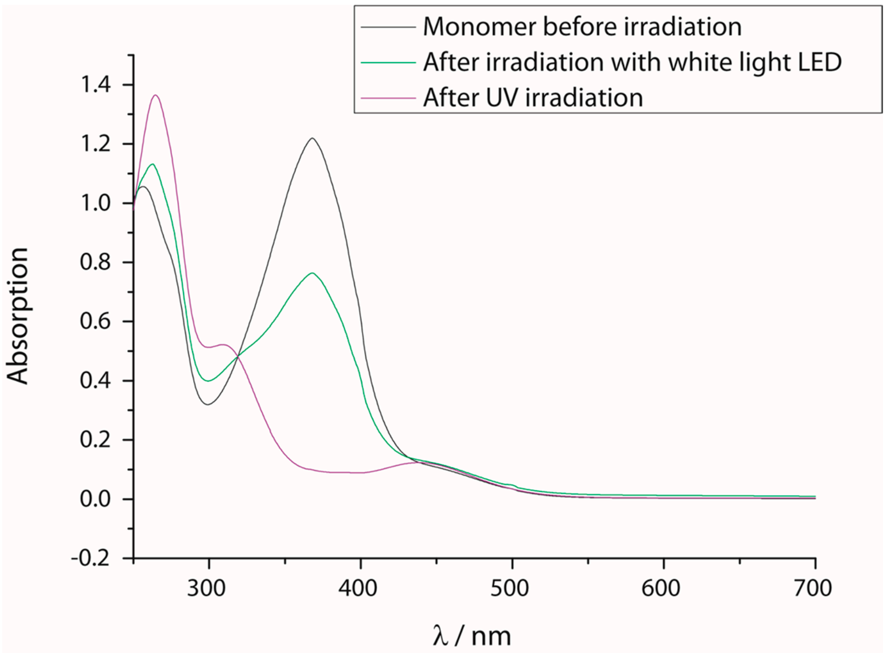

Figure S2. UV–vis spectra of the monomer before irradiation and after irradiation with the white light LED as well as with UV light.

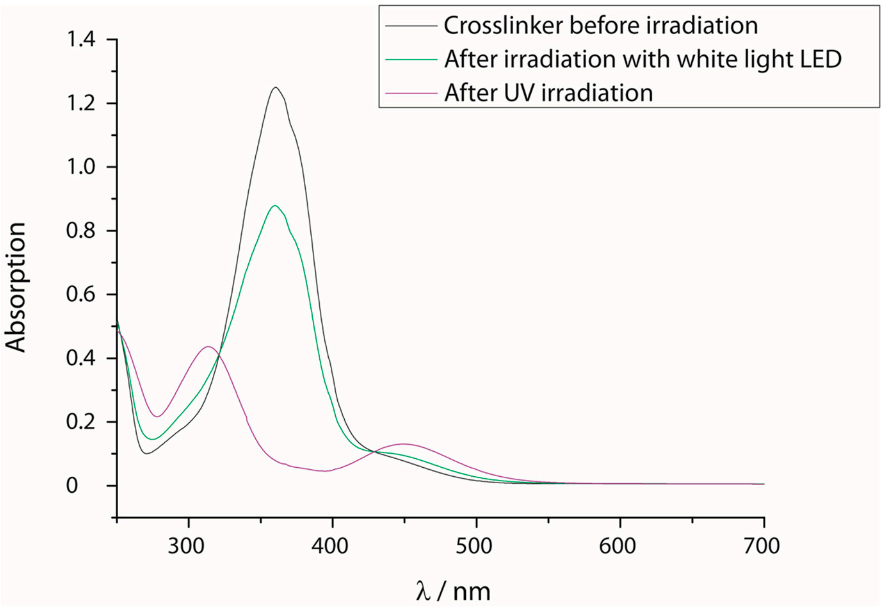

Figure S3. UV–vis spectra of the crosslinker before irradiation and after irradiation with the white light LED as well as with UV light.
